# Knowledge, attitudes, and practices of adults in the Kingdom of Saudi Arabia regarding *Helicobacter pylori*-induced gastric ulcers, cancers, and treatment

**DOI:** 10.25122/jml-2023-0536

**Published:** 2024-05

**Authors:** Mohammed Attieh Alzahrani, Saeed Jarallah AlQahtani, Meshari Saad Alqahtani, Hatem Mostafa Asiri, Abdulaziz Mohammed Abudasir, Khalid Talab Alshahrani, Ahmed Saad Al Zomia

**Affiliations:** 1 Department of Medicine, College of Medicine, King Khalid university, Abha, Saudi Arabia; 2 College of Medicine, King Khalid University, Abha, Saudi Arabia

**Keywords:** *Helicobacter pylori*, awareness, knowledge, attitudes, practices, community, Saudi Arabia

## Abstract

*Helicobacter pylori* (HP) infection presents a significant threat to global health with serious associated morbidities. This study aimed to assess awareness, attitudes, and practices related to HP in the Kingdom of Saudi Arabia (KSA) through a survey-based cross-sectional study involving 2,541 respondents. We used a structured online questionnaire to gather data on personal and sociodemographic characteristics, as well as HP-related knowledge, attitudes, and practices. The survey was distributed through various social media platforms. The results revealed that 59.4% of respondents demonstrated good knowledge about HP, with a mean knowledge score of 3.7 ± 1.0 out of 5. Knowledge gaps were particularly evident regarding the contagiousness and transmission modes of HP. The mean attitude score was 12.2 ± 2.2 out of a maximum score of 15. In total, 37.6% of respondents reported ever being tested for HP, with 54.2% testing positive. Among those treated for HP, only 79% received antibiotic therapy and 37.8% received acid-reducing medications. Knowledge levels were significantly higher among younger and highly educated respondents (*P* < 0.001), and respondents with higher knowledge scores also had higher attitude scores than those with lower knowledge scores (12.6 ± 2.0 vs. 11.6 ± 2.0, *P* < 0.001). Individuals who had undergone HP testing had significantly higher knowledge levels than those who did not (62.3 vs. 57.8, *P* = 0.024). These findings underscore the urgent need for raising the population’s awareness regarding the risks, prevention, and management of HP infection through targeted educational strategies.

## INTRODUCTION

*Helicobacter pylori* (HP) is a Gram-negative, microaerophilic, spiral-shaped bacterium that colonizes the gastric mucosa in humans. In addition to its high prevalence worldwide, HP is a significant risk factor for various gastrointestinal diseases, including chronic gastritis, peptic ulcers, gastric adenocarcinoma, and mucosa-associated lymphoid tissue (MALT) lymphoma [1,2]. HP infection has become a global public health concern [3]. The infection is mainly transmitted through direct person-to-person contact and waterborne transmission [2].

The worldwide prevalence of HP infection is estimated to 44.3%, developing countries exhibiting higher infection rates than developed countries (50.8% vs. 34.7%) [4]. The infection rate reported by several studies conducted in different regions in the Kingdom of Saudi Arabia (KSA) ranges from 10.2 % to 45.6%, varying by the studied population’ age, physical status, socioeconomic status, geographical location, and laboratory assessment method [1,5,6].

The World Health Organization (WHO) has classified HP as a group I carcinogen. HP causes gastric cancer, which is considered the sixth most common malignant tumor and the fourth most common cause of cancer-related deaths worldwide. In 2020, there were more than one million cases of gastric cancer associated with HP infection worldwide [1,7]. Given the close association between HP and gastric cancer, a meta-analysis showed that eradication of HP can reduce the incidence and mortality of gastric cancer [8,9].

Several national and international studies have highlighted the poor awareness of the population regarding HP [10–14]. Population awareness level affects the adoption of a healthy lifestyle and healthy behaviors. It was previously reported that a community’s level of awareness and health literacy is correlated with positive health-seeking behaviors such as HP screening and participation in health education campaigns [11,14,15].

Given that HP is highly prevalent in the KSA and a risk factor for gastric cancer, improving awareness levels regarding HP infection could influence risk perceptions, and improve health-seeking behaviors and people’s medical decisions. Such influences could be regarded as a primary preventive strategy for the serious consequences of HP.

Understanding public knowledge about HP in the KSA would provide baseline data regarding awareness, attitudes, and practices related to HP infection among the Saudi population, essential for tailoring appropriate educational interventions and awareness-raising activities and strategies. The aim of this study was to assess the general population’s knowledge attitudes, and practices related to HP infection in a representative sample of the Saudi population.

## MATERIAL AND METHODS

### Study design, setting, and population

We conducted a descriptive cross-sectional study from May 2023 to December 2023 among residents of different regions of the KSA: Riyadh, Assir, Eastern Region, Mecca, Al-Qassim, Hail, Tabuk, Al-Madinah Al-Monawarah, Najran, Al-jawf, Al-Bahah, Jazan, and Northern Border Region. The study population included male and female residents of the aforementioned regions who were aged above 18 years and were able to use the internet and social media platforms. Participants younger than 18 years and those with poor digital literacy or not able to use the internet were excluded from the study.

Participants were recruited using non-probability convenience and snowball sampling techniques via various social media platforms. Announcements and study objectives were posted alongside the study questionnaire's Google Form link on platforms such as Facebook groups, WhatsApp groups, X, and Telegram. Participants who expressed interest in joining the study received the questionnaire link and were encouraged to share it with their contacts.

### Study sample

A previous study conducted in the KSA identified a 54.9% awareness level related to HP among the study participants [16]. Based on these data, a precision of 5% at 95% confidence level and design effect of 2, we calculated a minimum required sample size of 764 individuals using Epi-Info 7 software. As the data were expected to be collected using an online questionnaire, a 40% non-response rate was considered, yielding a final minimum sample of 1,070 individuals. Until the end of the study, 2,541 individuals have completed the survey.

### Study instrument

After clearly explaining the purpose of the study, individuals who accepted to participate in the study were asked to fill in an online self-administered questionnaire in Arabic, consisting of four sections. The first section included sociodemographic characteristics such as age, sex, education, residence, and nationality. The second section included five questions with ‘Yes’ or ‘No’ answers and assessed the participants’ knowledge about HP, including type of the organism, transmission and contagiousness, risk of peptic ulcer and gastric cancer associated with HP infection, and the use of antibiotics as an effective treatment for HP infection. Correct answers were scored with 1 point and incorrect answers with 0 points, yielding a maximum score of 5. The knowledge scores were further categorized into low and high knowledge categories based on a 60% cut-off point. Scores below 3 were labelled as ‘poor knowledge’, and scores equal to or above 3 were labelled as ‘good knowledge’. The third section assessed attitudes and perceptions of respondents towards HP infection using three questions related to concern about the risks of HP infection, perceived seriousness of the condition in the KSA, and recognition of the importance of educating KSA residents about HP. The questions were answered using a 5-point Likert scale (‘Strongly agree’, 5 points; ‘Agree’, 4 points; ‘Neutral’, 3 points; ‘Disagree’, 2 points; ‘Strongly disagree’, 1 point), higher scores representing a positive attitude and lower scores representing a negative attitude. The maximum attitude score was 15. For reliability assessment, the Cronbach’s alpha coefficient for the questions assessing the respondents’ attitude towards HP was 0.633. The fourth section included questions about experiences and practices linked to HP infection. Participants were asked if they had ever been tested or screened for HP, the results of those tests, and whether they had ever received treatment for HP. In addition, they were asked about the type of treatment received and how often they discussed HP infection and its risks with their social circle.

The questionnaire was revised by experts at the medical college of King Khalid University to assess the face validity of the questionnaire, using structural and content validity. A pilot test was conducted among 50 respondents to assess the clarity of the questions and the time needed to fill in the questionnaire. The language of the questionnaire was slightly revised based on feedback from the pretest, and those who participated in the pretest did not fill in the final questionnaire.

### Statistical analysis

Statistical analysis was performed using SPSS v.24.0 (IBM Corp). Categorical variables were reported as proportions or percentages, and numerical variables were represented as mean ± s.d. when applicable. For bivariate analysis, Pearson’s chi-squared test was used for categorical variables, and the independent sample *t*-test was used to compare means of quantitative variables. All statistical tests used two-tailed tests and a *P* value of ≤0.05 was considered significant.

## RESULTS

### Participants’ personal and sociodemographic characteristics

A total of 2,541 individuals participated in the study. Their sociodemographic characteristics are presented in Table 1. The respondents’ age ranged from 18 to 65 years, over half of them were women (*n* = 1,572; 61.6%), and the majority (*n* = 2,291; 90.2%) were Saudi. More than a third of respondents (*n* = 950; 37.4%) were university students, and 1,029 (40.5%) completed university education and above. Residents of Mecca, Riyadh, and Assir regions represented 24.%, 23.7%, and 16.3% of the sample, respectively.

**Table 1:** Respondents’ personal and sociodemographic characteristics

Characteristic (*n* = 2,541)	Level	*n* (%)
**Sex**	Male	969 (38.1)
	Female	1,572 (61.9)
**Age**	18–25 years	1,168 (46.0)
	26–45 years	797 (31.4)
	46–65 years	576 (22.7)
**Nationality**	Saudi	2,291 (90.2)
	Non-Saudi	250 (9.8)
**Education**	Less than secondary	160 (6.3)
	Secondary	402 (15.8)
	University	950 (37.4)
	University and above	1,029 (40.5)
**Residence**	Mecca	633 (24.9)
	Riyadh	603 (23.7)
	Assir	413 (16.3)
	Al-Madinah Al-Monawarah	365 (14.4)
	Eastern	220 (8.7)
	Other	307 (22.0)

### Knowledge about HP

The distribution of answers to the questions assessing knowledge about HP is presented in Table 2. The average knowledge score was 3.7 ± 1.03. Of the 2,541 respondents, 1,030 (40.5%) and 1,511 (59.5%) had poor and good knowledge of HP, respectively.

**Table 2:** Knowledge about HP among respondents

Characteristic (*N* = 2,541)	Level	*n* (%)
**HP is a type of bacteria**	Correct	2,256 (88.8)
	Incorrect	285 (11.2)
**HP infection could cause peptic ulcer**	Correct	2,181 (85.8)
	Incorrect	360 (14.2)
**HP infection increases the risk of gastric cancer**	Correct	1,888 (74.3)
	Incorrect	653 (25.7)
**HP infection is contagious and can be transmitted from one person to another**	Correct	1,070 (42.1)
	Incorrect	1,471 (57.9)
**Antibiotics are important for the treatment of HP**	Correct	1,988 (78.2)
	Incorrect	553 (21.8)
**Total knowledge**	Good knowledge	1,511 (59.5)
	Poor knowledge	1,030 (40.5)
**Total knowledge score, mean (s.d.)**	3.7 (1.03)	

Overall, 2,256 respondents (88.8%) knew that HP is a bacterial infection, 2,181 (85.8%) knew that HP is associated with increased risk of peptic ulcer, and 1,988 (78.2%) were aware that antibiotics are an important component of HP therapy. Despite the high awareness level among the study population, only 1,070 respondents (42.1%) knew that HP is contagious and can be transmitted from an infected person to a healthy one. Based on the cut-off point of 3 out of 5 points, more than half of the sample (*n* = 1,511; 59.5%) showed good knowledge.

### Attitudes and perceptions towards HP

**Figure 1:**
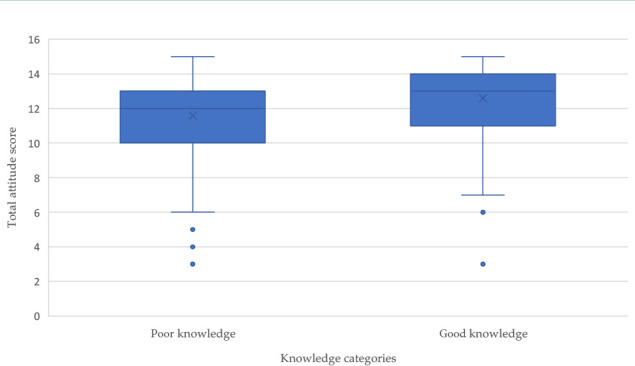
Box plot for attitude scores among knowledge categories

The distribution of answers to questions assessing attitudes and perceptions toward HP infection is presented in Table 3. Most of respondents held a positive attitude towards HP, with a mean attitude score of 12.19 ± 2.15 out of the maximum 15. Most respondents (*n* = 2,260; 88.9%) believed that people in the KSA should be educated about HP infection, its prevention, and associated risks. More than two thirds (*n* = 1,767; 69.6%) were concerned about the health risks of HP, and 1,630 (64.1%) regarded HP as a significant public health problem in the KSA. The box plot analysis of knowledge and attitude scores showed that high knowledge levels were associated with high attitude scores (Figure 1).

**Table 3:** Attitudes and perceptions towards HP

Characteristic (n = 2,541)	Strongly agree/ Agree *n* (%)	Strongly disagree/ Disagree *n* (%)	Neutral *n* (%)	Mean (s.d.)
**I am concerned about the health risks of HP**.	1,767 (69.6)	245 (9.6)	529 (20.8)	3.93 (1.02)
**I believe that HP infection is a significant public health issue in the KSA**.	1,630 (64.1)	280 (11.1)	631 (24.8)	3.81 (1.02)
**I believe that people in the KSA should be educated about HP infection, prevention, and its potential health risks**.	2,260 (88.9)	62 (2.4)	219 (8.6)	4.45 (0.78)
**Total attitude score, mean (s.d.)**	12.19 (2.15)			

### Experiences and practices related to HP

The distribution of answers regarding the respondents’ experiences and practices related to HP are presented in Table 4. Despite the high knowledge and attitude scores obtained in the first part of the questionnaire, nearly one third of respondents (*n* = 995; 37.6%) were tested for HP, and more than half of those tested (*n* = 518; 54.2%) had positive results, representing approximately 20% of the entire study population (not illustrated in the table). Those who tested negative (*n* = 334; 35%) represented 13.2% of the study population (not illustrated in the table). Of the tested respondents, 103 (10.8%) did not remember the result of their test.

**Table 4:** Experiences and practices related to HP

Characteristic (*n* = 2,541)	Level	*n* (%)
**Ever tested for HP**	Yes	955 (37.6)
	No	1,586 (62.4)
**Result of the HP test?** **(*n* = 955)**	Positive	518 (54.2)
	Negative	334 (35.0)
	Do not remember	103 (10.8)
**Ever received treatment for HP?**	Yes	848 (33.4)
	No	1,693 (66.6)
**Type of HP treatment received (*n* = 756)**	Antibiotics	597 (79.0)
	Acid-reducing medications	286 (37.8)
	Other	149 (19.7)
**Frequency of discussing HP infection and its associated health risks with family, friends, or community members**	Frequently	376 (14.8)
	Sometimes	904 (35.6)
	Rarely	786 (30.9)
	Never	475 (18.7)

In total, 848 respondents (33.4%) received treatment for HP, of which 597 (79.0%) received antibiotic treatment, 286 (37.8%) received antacids or acid reducing medications, and 149 (19.7%) received other treatment.

Raising the issue of HP infection in day-to-day discussions with family, friends, or community members was frequent among 376 respondents (14.8%), whereas 904 respondents (35.6%) reported that they sometimes discuss this issue. Nearly one-fifth of participants (475; 18.7%) never discussed HP with their surroundings.

### Factors associated with knowledge about HP

The factors associated with knowledge about HP are presented in Table 5. We found that knowledge level was inversely related to respondents’ age, good knowledge levels being higher among young individuals and decreasing with age. Nearly two-thirds of young respondents aged 18–25 (*n* = 742; 63.5%) had good knowledge compared to 293 respondents (50.9%) of 46–65 years (*P* < 0.001). Furthermore, the proportion of respondents with good knowledge increased significantly with the level of education, being 47.5% among respondents with less than secondary education compared to 60.1% among respondents with university education and above (*P* < 0.001). Sex and nationality did not seem to be associated with the level of knowledge.

**Table 5:** Participants’ knowledge regarding HBP and associated factors

Characteristic (*n* = 2,541)		Good knowledge	Poor knowledge	*P* value
**Sex**	Male	585 (60.4)	384 (39.6)	0.480^a^
	Female	926 (58.9)	646 (41.1)	
**Age**	18–25 years	742 (63.5)	426 (36.5)	<0.001^*a^
	26–45 years	476 (59.7)	321 (40.3)	
	46–65 years	293 (50.9)	283 (49.1)	
**Nationality**	Saudi	1,359 (59.3)	932 (40.7)	0.684^a^
	Non-Saudi	152 (60.8)	98 (39.2)	
**Education**	Less than secondary	76 (47.5)	84 (52.5)	<0.001^*a^
	Secondary	217 (54.0)	185 (46.0)	
	University	600 (63.2)	350 (36.8)	
	University and above	618 (60.1)	411 (39.9)	
**Ever tested for HP**	Yes	595 (62.3)	360 (37.7)	0.024^*a^
	No	916 (57.8)	670 (42.2)	
**Ever treated for HP**	Yes	525 (61.9)	323 (38.1)	0.079^a^
	No	986 (58.2)	707 (41.8)	
**Frequency of discussing HP**	Frequently	253 (67.3)	123 (32.7)	<0.001^*a^
	Sometimes	557(61.6)	347 (38.4)	
	Rarely	453 (57.6)	333 (42.4)	
	Never	246 (52.2)	227 (47.8)	
**Total attitude score towards HP, mean (s.d.)**		12.6 (2.0)	11.6 (2.0)	<0.001^*b^

a chi-squared test; ^b^ independent sample t-test; * statistically significant

We also found an association between knowledge and attitude scores, as respondents with good knowledge scores tended to have significantly higher attitude scores compared to those with low knowledge (12.6 ± 2 vs. 11.6 ± 2, *P* < 0.001).

Regarding the respondents’ experience and practices regarding HP infection, those who were previously tested for HP had significantly higher scores than those who did not (61.9 % vs. 58.2%; *P* = 0.024). A similar trend was observed between respondents who discussed HP frequently with family, friends, or community members and those who did not (67.3% vs. 52.2%; *P* < 0.001).

## DISCUSSION

Understanding people’s awareness, attitudes, and practices related to HP infection can help develop tailored national prevention and screening strategies. In our study population, about one-fifth of participants tested positive for HP, indicating the considerable burden of HP infection among the Saudi population. More than half of the study participants (59.5%) had high awareness levels regarding HP, and most of them showed a positive attitude regarding the seriousness of HP infection. However, the proportion of respondents who had undergone HP testing or screening was much lower at 37.6%.

### Knowledge about HP

Although more than 75% of respondents knew the correct answer for most of the questions, only 42.1% knew that HP infection is contagious and can be transmitted from one person to another. Most of them knew that HP is caused by bacterial infection and increases the risk of peptic ulcer and gastric cancer. They were also aware of the importance of antibiotics in the treatment of HP. In general, we found that the general population had average to good knowledge.

The results of this study can be explained in part by two recent cross-sectional studies conducted in the KSA, which found that study participants were substantially influenced by information provided through social media platforms [17,18]. Similar knowledge levels were reported by another study conducted in the Al-Ahsa region of the KSA[16]. Despite some differences in the questions used to assess the level of knowledge, similar mean awareness levels were reported in other population-based studies in the KSA [5,13,19] and Croatia [12]. However, other studies reported lower awareness levels among cohorts from the KSA [16], China [11,15], and the UAE [20], with only 24.6% of respondents reporting that they had heard about HP in the study conducted in the UAE. Similarly to our findings, studies from China and the UAE revealed a knowledge gap regarding the contagiousness and modes of transmission of the infection compared to other areas of knowledge. Future educational efforts should emphasize the modes of transmission of HP infection and risky behaviors associated with it.

In addition to differences in the questions used to assess respondents’ knowledge among different studies, the discrepancy in awareness levels could be attributed to the type of study population and the data collection methods used. Studies reporting higher knowledge levels predominantly relied on online surveys, which tend to attract more knowledgable internet users. By contrast, studies reporting lower knowledge levels mainly collected data through interviews, in which less knowledgable and lower-educated individuals were more represented.

Awarness of HP was found to be influenced by certain sociodemographic characteristics. Our study revealed that older and less educated respondents had lower knowledge levels. Similar results were reported by studies conducted in Croatia [12], China [15], and Cameroon [21]. However, unlike the Chinese and Cameroon studies, our study did not find the respondents’ sex to influence awareness levels. In addition, a recent study in the KSA found no association between the participants’ sociodemographic characteristics and their knowledge about HP [13]. Contradicting our findings, another study in the KSA reported that the only sociodemographic factor associated with high awareness level was participants’ age, with older respondents showing higher awareness [16]. The difference could be attributed to differences in study populations, settings, and methdologies. For example, the study conducted by Al Ghadeer *et al*. included university students, with the oldest age group being in their thirties, whereas our study was population-based and included participants up to 65 years old.

Meanwhile, younger people and highly educated individuals showed higher awareness levels. This could be attributed to the fact that younger people, who are often still students, as well as highly educated individuals, have access to reliable information sources. A recent study in the KSA also reported that educational level was significantly associated with HP awareness [19]. This may be explained by the fact that the Ministry of Health in the KSA has an electronic media interface that publishes information on health issues through an awareness platform accessible to students [13]. These findings highlight the need for future educational efforts to target older adults and less educated individuals through different strategies and media, not solely internet-based or online education.

It has been previously noted that undergoing a screening test or an investigation for a condition makes people more informed about it [11,13]. The current study confirmed this, showing that people who underwent HP testing had significantly higher levels of knowledge than those who did not. However, having a positive result and undergoing treatment for the condition did not seem to influence the knowledge level. This might be because most people who had an HP test, regardless of their test results, had higher knowledge. It appears that the experience of being tested, rather than the test results themselves, influenced participants’ knowledge. This may indicate that healthcare providers do not devote enough effort to educate patients about the disease, its transmission, or its complications. Future studies should focus on healthcare professionals’ practices regarding HP in the KSA.

### Attitudes towards HP

Although nearly two-thirds of the respondents were concerned about the health risks of HP and perceived it as a major public health concern in the KSA, a much higher percentage (88.9%) emphasized the increasing need for education about HP. Although we found a significant association between the respondents’ knowledge and attitudes, this finding suggests that people might not be satisfied with their current knowledge about HP and with their sources of information, and require more reliable and trusted information on the topic.

According to the results of our study, people tend to discuss HP infection with family and friends. The reported need for education and frequent discussion of the topic suggest HP is regarded as a prevalent and serious condition in the KSA that demands serious action. Future studies should deeply investigate the informational needs of people in the KSA and their preferred and trusted sources of health information.

### Practices related to HP

Although more than one third of respondents had previously undergone HP testing and half of them (518 participants) tested positive, a higher number (848 participants) reported receiving HP treatment. Studies suggest that negative test results may occur in up to 56% of cases, especially during the early phase of infection [22–24], indicating that nearly half of individuals with negative results may still require HP treatment. Our study showed that the proportion of respondents who received HP treatment was higher than that of those who tested positive, raising questions about how physicians decide whom to treat and when to prescribe HP medications. In addition, despite the fact that antibiotic therapy is a main component in HP treatment, only 79% of respondents treated for HP received antibiotics, and only 37.8% received acid-reducing medications. These findings highlight potential malpractices related to the diagnosis and management of HP infection in the KSA and raise concerns about the availability and adherence to HP management guidelines. There is an urgent need to investigate knowledge and practices related to HP among physicians in the KSA, as well as patients’ compliance with treatment, both quantitatively and qualitatively. Qualitative research would provide deeper insights into physicians’ practices regarding the diagnosis and management of HP, as well as patients’ treatment-seeking behaviors and compliance with treatment.

### Study limitations

To our knowledge, this is the first study to assess HP awareness across all regions of the KSA. However, the study has several limitations. First, it did not assess knowledge of preventive measures for HP, attitude towards screening, and respondent behaviors. Second, the representativeness of the study population may be questioned, as the study included a convenience sample of participants familiar with using computers and the internet, who had internet access and a certain degree of digital literacy. In addition, the self-selection of participants could also affect representativeness, as individuals who voluntarily chose to participate might have different characteristics regarding their knowledge or previous HP experience compared to those who refused to participate. Third, data on HP testing, test results, and treatment regimens relied on self-reporting, which may be subject to recall bias. Finally, the lack of a global, validated questionnaire to assess knowledge, attitudes, and practices regarding HP infection limits the comparability of our results with other studies.

## CONCLUSION

Although the HP awareness level was acceptable in the current study, important knowledge gaps were identified. Participants expressed a clear need for more education on HP, and our findings suggest that everyone in the KSA, regardless of age, educational level, or degree of digital literacy, needs to be educated about HP.

Study participants indicated different informational needs, including preventive measures, modes of transmission, diagnostic modalities, and treatment options, with an emphasis on the importance of adherance and compliance to treatment to minimize antibiotic resistance. In addition, doctors should be continously updated with recent diagnostic and management guidelines. They should also be advised about the importance of educating patients about dietary habits, modes of transmission, and related risks and complications associated with HP.

The high incidence of HP calls for a national screening plan to detect the infection early and initiate treatment, aiming to decrease the incidence of HP-related complications such as peptic ulcer and gastric cancer. Further research is needed to gain deeper insights into physicians’ practices regarding the diagnosis and management of HP, as well as patients’ treatment-seeking behaviors and compliance with treatment.

## Data Availability

Further data is available from the corresponding author upon reasonable request.
